# Prenatal diazepam exposure impairs maternal caregiving behaviors in rats: Roles of GABAARα1 downregulation, serotonin depletion, and corticosterone dysregulation

**DOI:** 10.1016/j.toxrep.2025.102186

**Published:** 2025-12-13

**Authors:** Yasaman Moin, Samira Khayat, Hamed Fanaei

**Affiliations:** a School of Medicine, Zahedan University of Medical Sciences, Zahedan, Iran; b Pregnancy Health Research Center, Zahedan University of Medical Sciences, Zahedan, Iran; c Cellular and Molecular Research Center, Research Institute of Cellular and Molecular Sciences in Infectious Diseases, Zahedan University of Medical Sciences, Zahedan, Iran; d Department of Physiology, School of Medicine, Zahedan University of Medical Sciences, Zahedan, Iran

**Keywords:** Maternal behavior, Diazepam, Pregnancy, Serotonin, Corticosterone, GABA receptor

## Abstract

This study investigated effects of prenatal exposure to diazepam on maternal and caregiving behaviors in rats postpartum.Twenty-four female rats were randomly divided into two groups: diazepam group and control group. Diazepam was administered during, and maternal behaviors were observed and recorded after delivery. Serum corticosterone levels during pregnancy, GABAARα1 expression, and serotonin and BDNF concentrations were measured in hippocampus and prefrontal cortex of the dams. The results showed that mothers exposed to diazepam exhibited a significant reduction in self-grooming (p = 0.0016), nursing (p < 0.0001), and nest-building behaviors (p < 0.0001) compared to the control group. Additionally, diazepam group showed fewer instances of pup retrieval (p = 0.0032) and licking (p = 0.0019). A significant increase in the latency to retrieve pups was observed in the diazepam group (p < 0.0001). The findings demonstrate a significant decrease in GABAARα1 mRNA expression within the prefrontal cortex (P = 0.0023) and hippocampus (P = 0.0138) of diazepam-treated group compared to the control group. Dams in the diazepam group exhibited significantly lower serum corticosterone levels at gestational day 20 (p = 0.0288) and postnatal day 1 (p = 0.0009) compared to the control group. Additionally, serotonin concentration in the prefrontal cortex (p = 0.0036) was significantly reduced in the diazepam group relative to controls.The present study demonstrated that prenatal diazepam exposure significantly impaired maternal caregiving behaviors in rats. These behavioral deficits were associated with disrupted serum corticosterone levels, diminished prefrontal serotonin concentrations, and reduced GABAARα1 mRNA expression in the prefrontal cortex and hippocampus. The findings suggest that diazepam interferes with neurochemical pathways critical for maternal motivation, potentially weakening maternal-infant bonding.

## Highlights

• Prenatal diazepam impaired rat maternal care and altered neurochemical markers (GABAARα1,serotonin,corticosterone) postpartum.
• Diazepam reduced dam self-grooming, nursing, nest-building, pup retrieval, and licking, compromising maternal care.
• It lowered prefrontal GABAARα1 mRNA, reduced serum corticosterone, and decreased prefrontal serotonin.
• Deficits link disrupted stress/mood neurochemistry to impaired maternal motivation and infant bonding.

## Graphical Abstract

**Figure:**
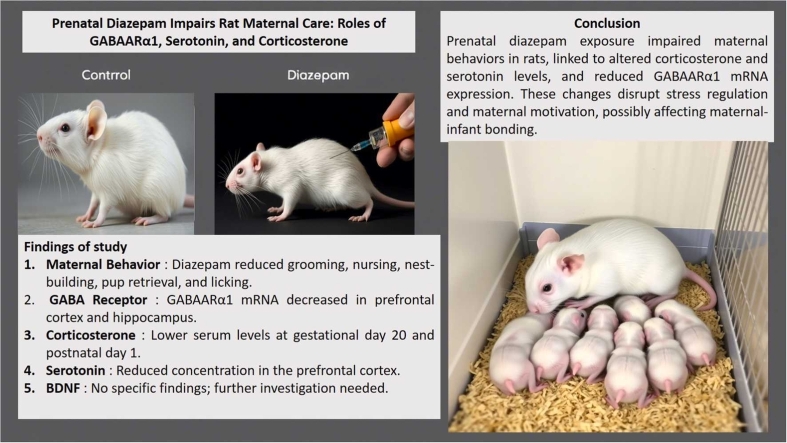

## 1 Introduction

Pregnancy is one of the most critical and sensitive periods in a woman’s life, characterized by significant physical, social, and psychological changes that can profoundly impact both maternal and neonatal health in subsequent stages [1]. Sleep disturbances, such as insomnia, are among the most common complaints during pregnancy, often manifesting as reduced sleep duration and diminished sleep quality. Additionally, anxiety is another prevalent condition experienced by many pregnant women. Initially, this anxiety is often related to concerns about the baby, such as potential developmental abnormalities. Over time, it may shift to focus on the mother herself. If anxiety persists for more than three weeks, it can pose serious risks to both maternal and fetal health [1]. Pharmacological intervention is one approach to managing these issues. Benzodiazepines, a class of drugs commonly used to control anxiety and induce sleep, are frequently prescribed [2]. Diazepam, also known as Valium, is a fast-acting and long-lasting benzodiazepine typically used to treat anxiety disorders, provide short-term relief of anxiety symptoms, manage alcohol withdrawal, control acute seizures, and address insomnia [2]. However, diazepam is classified as a pregnancy category D drug, indicating that its use is associated with an increased risk of congenital malformations, preterm birth, low birth weight, and other neurodevelopmental abnormalities [3]. Diazepam readily crosses the placental barrier, and its use during pregnancy may lead to neonatal withdrawal syndrome immediately after birth [3]. Given the potential risks of fetal abnormalities, dependency, and withdrawal, the prescription and use of diazepam during pregnancy remain controversial. Substance misuse during pregnancy and lactation can severely compromise maternal and neonatal health, particularly when the substances affect the central nervous system [4]. Such misuse can disrupt maternal behavior and caregiving, leading to adverse outcomes for the fetus [5]. Childbirth marks a dramatic transitional moment in the mother-infant relationship. From this point onward, the intimate anatomical and physiological connection between mother and child is replaced by breastfeeding and other behavioral interactions, underscoring the importance of maternal behavior [5]. Maternal behavior, a subset of parental behavior, is defined as any action performed by an adult member of a species toward an immature member to ensure the latter’s survival until maturity [6].

The laboratory rat provides a robust model for investigating the neurobiological substrates of maternal behavior. During pregnancy, the dam undergoes significant endocrine changes, including rising levels of estradiol and prolactin and a pre-parturition drop in progesterone, which prime the brain for the immediate onset of maternal care after delivery [7], [8]. This care is characterized by a highly stereotyped and motivated repertoire including nest-building (creating a sheltered environment for the litter), pup retrieval (gently carrying scattered pups back to the nest), nursing (assuming a characteristic crouching posture to allow pups to feed), and anogenital licking (which stimulates pup elimination and provides tactile stimulation) [9]. These behaviors are critically dependent on a network of brain regions including the medial preoptic area, the mesolimbic dopamine pathway, and the prefrontal cortex, which integrates executive function with motivational states [10].

The transition to motherhood also involves a carefully orchestrated stress response, mediated by the hypothalamic-pituitary-adrenal (HPA) axis and corticosterone release in rats, which is essential for providing the energy required for parturition and lactation [9], [11]. Furthermore, neurotransmitters like serotonin (5-HT) and GABA are deeply implicated in modulating mood, anxiety, and the execution of compulsive, care-oriented behaviors.

While previous research has explored the adverse effects of various substances on maternal behavior during pregnancy, the specific impact of diazepam on maternal behavior remains underexplored. Given the critical role of maternal behavior in child development and future outcomes, it is essential to investigate factors that may influence it. This study aims to examine the effects of diazepam on maternal behavior in a rodent model, addressing a significant gap in the literature.

## 2 Materials and methods

After obtaining ethical approval and the necessary permits (ethical code: IR.ZAUMS.AEC.1402.001), the study commenced. Twenty-four female Sprague-Dawley albino rats, weighing between 200 and 220 g, were used and divided into two groups of twelve. The sampling method was simple random sampling, and the animals were randomly assigned to the groups. To induce pregnancy, two female rats were housed with one male rat in a single cage. Starting from the next day, vaginal smears were taken from the female rats to detect sperm [12]. Female rats that tested positive were transferred to separate cages in pairs. All animals had free access to food and water and were maintained under a 12-hour light/dark cycle at a temperature of 22 ± 2°C. Among the pregnant rats, 12 females were randomly selected for diazepam injection.

Diazepam (Caspian, Co) was administered at a dose of 1.25 mg/kg/day subcutaneously from GD14 to GD20. This low-to-moderate dose was selected to model therapeutic anxiolytic exposure in humans while avoiding sedative or overtly toxic effects in the dams [13], [14]. The treatment window targets a critical period of fetal brain development, including synaptogenesis and the maturation of GABAergic and monoaminergic systems, allowing for the investigation of specific neuroteratological effects on pathways relevant to maternal behavior [13], [14]. The control group received physiological saline on the same schedule.

On the second day postpartum, the animals and their pups were transferred to a larger cage for testing [9]. The floor of the cages was lined with wood shavings. Using gloves, pups were randomly scattered in the cage. A camera was installed above the cage to record maternal behaviors for 60 min. The number and duration of pup retrievals, the number and latency to initiate pup retrieval, the number and duration of pup licking (body and genital area), the number and duration of nest-building, the number and duration of nursing, and the number and duration of self-grooming were observed and recorded [9].

### 2.1 Measurement of GABAARα1 mRNA Expression via Real-Time Quantitative PCR (RT-qPCR)

To assess GABAARα1 mRNA levels in rat hippocampal and prefrontal cortex tissues, real-time quantitative PCR (RT-qPCR) was conducted using the following protocol. Hippocampal and prefrontal samples were collected immediately after maternal behavior evaluations and preserved in TRIzol solution (Invitrogen, Shanghai, China). Total RNA was isolated using the TRIzol-based extraction kit, adhering to the manufacturer’s guidelines. RNA quality and concentration were evaluated via spectrophotometric analysis, with absorbance ratios at 260/280 nm confirming purity. Reverse transcription of RNA into complementary DNA (cDNA) was performed with a Qiagen Reverse Transcription kit (USA), employing standardized incubation conditions to ensure synthesis efficiency. Gene-specific primers for GABAARα1 were designed using Primer5 software and synthesized by Shanghai Sangon Biotech (China). The primer sequences were:

• Forward: 5′-AGCCGAATGCCCCATGCACT-3′
• Reverse: 5′-CAACCACTGAGCGGGCTGGC-3′

For RT-qPCR, reactions were initiated with a 30-second predenaturation at 95°C, followed by 40 cycles of denaturation (95°C, 5 s) and annealing/extension (30 s). The housekeeping gene β-actin served as an internal control. Threshold cycle (Ct) values for GABAARα1 and β-actin were recorded, representing the cycle at which fluorescence surpassed the baseline. Relative mRNA expression was quantified using the 2 −ΔΔCt method, normalizing target gene Ct values to β-actin to account for variations in template quantity.

### 2.2. Corticosterone quantification

Serum corticosterone levels were assessed using a commercially available ELISA kit (Corticosterone ELISA Kit, ab108821, Abcam) following the manufacturer’s guidelines. Blood samples were collected via the tail vein under ketamine (100 mg/kg) and xylazine (10 mg/kg) anesthesia between 9:00–10:00 AM on gestational days (GD) 12, 16, and 20, as well as postnatal day 1 (PND1). Plasma was separated by centrifugation at 5000 rpm for 10 min and stored at −80°C until assayed. At the conclusion of the study, dams were euthanized under deep anesthesia induced by ketamine (100 mg/kg) and xylazine (10 mg/kg). The prefrontal cortex and hippocampal regions were promptly dissected and homogenized in phosphate-buffered saline (PBS) using a mechanical homogenizer. The homogenate was centrifuged at 3500 rpm for 15 min, after which the supernatant was collected and stored at −70°C until further analysis [12]. Serotonin and brain-derived neurotrophic factor (BDNF) concentrations in the prefrontal cortex and hippocampal samples were quantified using enzyme-linked immunosorbent assay (ELISA) kits (Zellbio GmbH, Germany), following the manufacturer’s protocols.

### 2.3 Statistical analysis

For data analysis, GraphPad Prism version 8.4 was used. Initially, the normality of data distribution was assessed using the Kolmogorov-Smirnov test. Since the data followed a normal distribution, independent samples T-tests were performed. A p-value of less than 0.05 was considered statistically significant.

A post-hoc power analysis was conducted using G*Power software (version 3.1.9.7) to determine the achieved statistical power of our key findings, given the sample size of n = 12 per group. The analysis was performed for a two-tailed independent samples *t*-test. The effect sizes (Cohen's d) observed for our primary behavioral outcome (e.g., pup retrieval latency) and primary molecular outcome (e.g., GABAARα1 expression) were substantial, both exceeding 1.5. With an alpha level (α) set at 0.05, this analysis confirmed that the study achieved a statistical power (1-β) greater than 0.95 for these endpoints. This indicates a less than 5 % probability of a Type II error and confirms that the sample size was adequate to detect the significant effects reported.

## 3 Results

### 3.1 Serum corticosterone levels

**Fig. 1:**
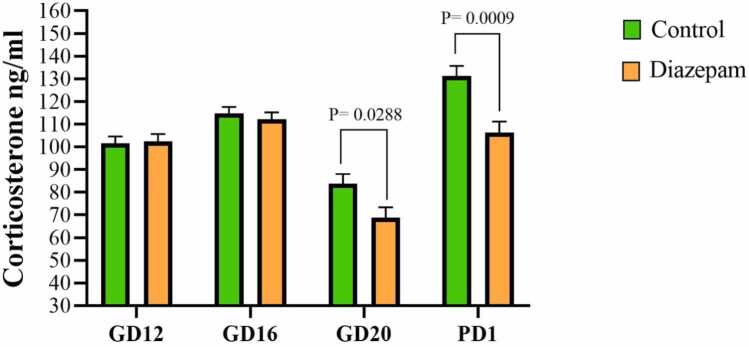
Serum corticosterone concentrations at different days during pregnancy and after pregnancy. GD12: Gestational day 12, GD16: Gestational day 16, GD20: Gestational day 20, PD1: Postnatal day 1.

Fig. 1 illustrates serum corticosterone concentrations at different time points during pregnancy (GD12, GD16, and GD20) and postpartum (PD1). A significant variation in corticosterone levels was observed across these time points, reflecting hormonal fluctuations associated with pregnancy and postpartum adaptation. Diazepam administration, initiated at GD14, significantly reduced corticosterone levels compared to the control group at GD20 (P = 0.0288) and PD1 (P = 0.0009), suggesting its potential role in modulating maternal stress regulation.

### 3.2 Serotonin and BDNF concentrations and GABAARα1 mRNA expression in the hippocampus and prefrontal cortex

**Fig. 2:**
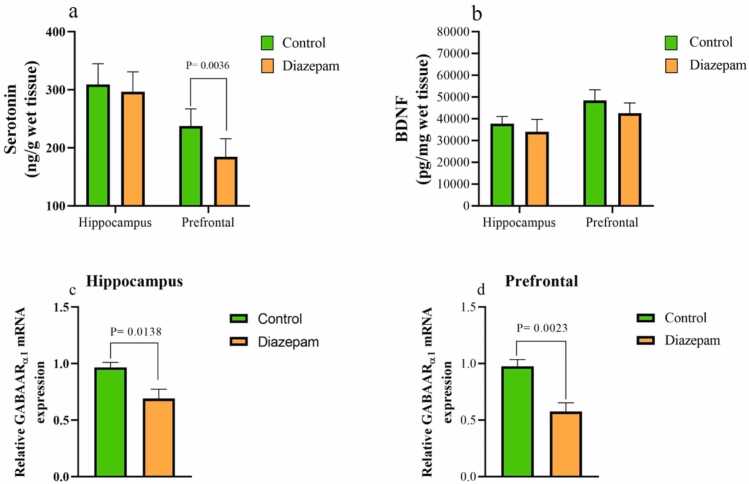
Serotonin and BDNF concentrations and GABAARα1 mRNA expression in the hippocampus and prefrontal cortex.

Fig. 2 presents serotonin and BDNF concentrations in the hippocampus and prefrontal cortex. The findings reveal significant differences in these neurochemical markers between the experimental and control groups. Diazepam administration led to a significant reduction (P = 0.0036) in serotonin levels in the prefrontal cortex, suggesting potential implications for maternal brain function and behavior. While BDNF levels in the diazepam group were lower in both the hippocampus and prefrontal cortex compared to controls, these differences were not statistically significant. The findings demonstrate a significant decrease in GABAARα1 mRNA expression within the prefrontal cortex (P = 0.0023) and hippocampus (P = 0.0138) of the diazepam-treated group compared to the control group (Fig. 2c-d).

### 3.3 Maternal behavior investigation

**Fig. 3:**
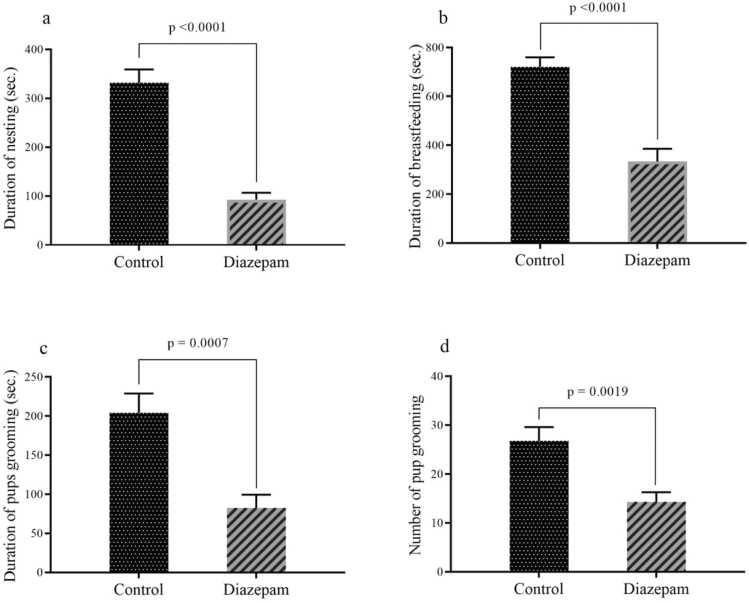
The results assess the endurance of maternal behaviors, including (a) nesting duration, (b) breastfeeding duration, (c) time spent grooming pups, and (d) the frequency of pup grooming. Data are presented as mean ± SEM.

As illustrated in Fig. 3a–d, the findings concerning the endurance of maternal behavior revealed that the diazepam-treated group exhibited significantly reduced mean durations of nesting (P < 0.0001), breastfeeding (P < 0.0001), and pup grooming (P = 0.0007), as well as a lower mean frequency of pup grooming episodes (P = 0.0019), compared to the control group. These results suggest that diazepam administration markedly impairs key aspects of maternal care, potentially reflecting disruptions in nurturing motivation or maternal responsiveness. The consistent reduction across multiple behavioral measures underscores the drug's pronounced inhibitory effect on maternal behavior.

**Fig. 4:**
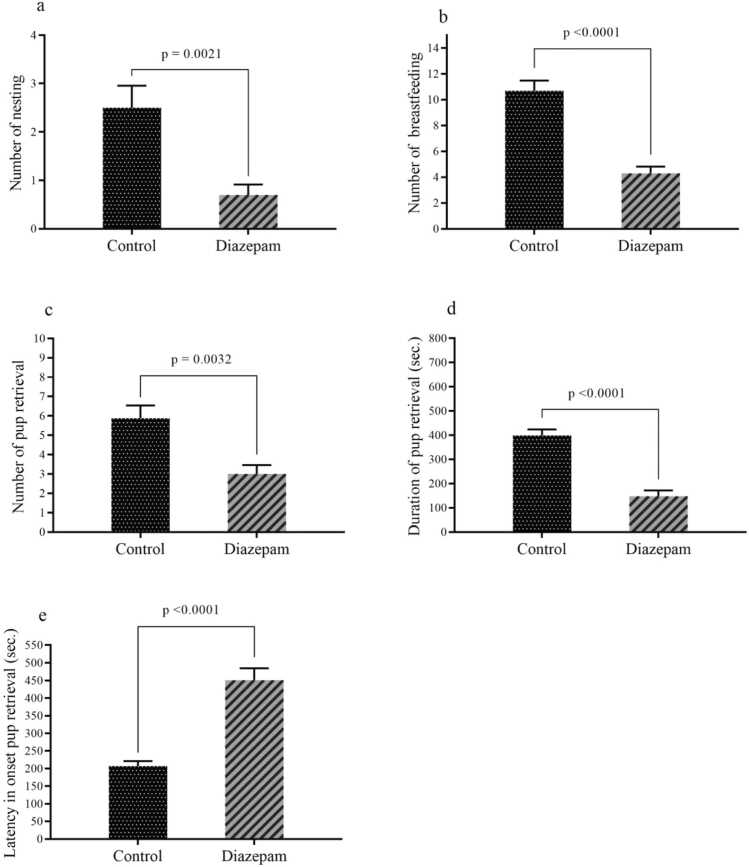
The results assess speed of integration of maternal behaviors, including (a) number of nesting, (b) number of breastfeeding, (c) number of pup retrieval, (d) duration of pup retrieval, and (e) latency in onset pup retrieval. Data are presented as mean ± SEM.

As depicted in Fig. 4a–e, various measures related to the speed of maternal behavior integration were significantly affected by diazepam administration. Specifically, the number of nesting events (P = 0.0021), instances of breastfeeding (P < 0.0001), and pup retrieval attempts (P = 0.0032) were significantly lower in the diazepam-treated group compared to the control group. Moreover, the latency to initiate pup retrieval was markedly prolonged (P < 0.0001), indicating a delay in maternal responsiveness. Conversely, the total duration of pup retrieval was significantly reduced (P < 0.0001). further highlighting impairments in maternal caregiving behaviors. These findings suggest that prenatal exposure to diazepam disrupts both the initiation and execution of essential maternal behaviors postpartum.

**Fig. 5:**
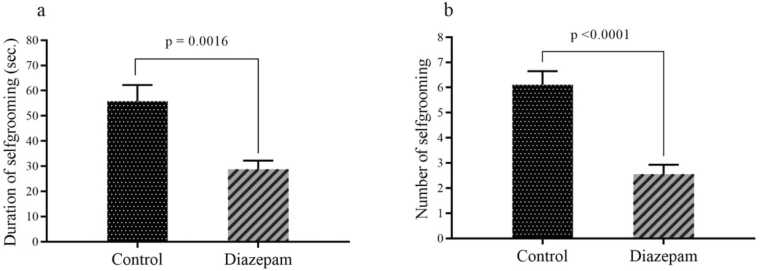
The results reflect emotional regulation (self-soothing and anxiety-related aspects) in maternal behavior, analyzed through two parameters: (a) total time spent self-grooming and (b) frequency of self-grooming episodes. Data are presented as mean ± SEM.

The results assessing emotional regulation (reflecting self-calming behaviors or anxiety levels) in maternal behavior revealed significant reductions in both (a) total self-grooming duration (P = 0.0016) and (b) frequency of self-grooming episodes (P < 0.0001) in the diazepam-treated group compared to controls (Fig. 5a, b). These marked decreases in self-directed grooming behaviors suggest that diazepam may impair typical self-soothing mechanisms or attenuate anxiety-related responses in dams, potentially disrupting their emotional balance during maternal care. The consistent suppression of both grooming duration and occurrence underscores a robust pharmacological effect on stress-related behavioral outputs.

## 4 Discussion

The profound disruption of maternal behaviors observed in this study following prenatal diazepam exposure underscores a complex interplay between pharmacological intervention, neuroendocrine regulation, and maternal caregiving. By integrating these findings with emerging mechanistic insights and clinical data, we propose a multifaceted model of diazepam’s impact on maternal behavior, emphasizing its implications for both maternal well-being and offspring development. The suppression of self-grooming in diazepam-treated dams reflects a critical divergence from typical stress-coping strategies. In rodents, self-grooming serves dual roles: it modulates anxiety via serotoninergic pathways and facilitates sensory resetting during maternal transitions [15]. Diazepam’s attenuation of this behavior may arise from its GABAergic potentiation, which dampens amygdala-driven stress responses [16]. However, this suppression could paradoxically impair maternal adaptability, as self-grooming is integral to maintaining hygiene and reducing infection risk during lactation [15]. The contrast with morphine-exposed dams, which exhibit heightened self-grooming [17], highlights drug-specific modulation of emotionality, where diazepam’s sedative effects may override adaptive stress behaviors. Nursing deficits further exemplify diazepam’s systemic impact. Lactation requires coordinated oxytocin and prolactin release, both of which may be disrupted by GABAergic overactivity [18], [19]. Reduced nursing not only limits nutritional and immunological transfer (e.g., immunoglobulins in colostrum) but also disrupts thermoregulatory contact, potentially exacerbating neonatal hypoglycemia and hypothermia [20], [21]. These findings align with human cohort studies linking benzodiazepine use during pregnancy to neurodevelopmental deficits, suggesting conserved mechanisms across species [22].

Nest-building impairments and prolonged pup retrieval latency likely stem from disrupted dopaminergic motivation circuits. The ventral tegmental area (VTA)-to-nucleus accumbens pathway, critical for goal-directed maternal behaviors [23], [24], may be inhibited by diazepam’s enhancement of GABAergic tone. This is consistent with fMRI research in humans, which indicates that reduced activation of the reward network in postpartum women with anxiety is linked to alterations in brain circuits involved in maternal care, empathy, motivation, emotional regulation, reward processing, and executive function [25]. These observations imply that benzodiazepines might diminish the significance of infant cues.

The marked reduction in corticosterone at GD20 and PD1 underscores diazepam’s suppression of the HPA axis. Corticosterone primes maternal care, cell proliferation of hippocampus and cognitive adaptations to heightened vigilance to newborn [26]. Its suppression may explain the delayed retrieval observed here, mirroring hypoactive HPA axis phenotypes seen in rodent models of maternal neglect. In these models, corticosterone is critical for pup retrieval, maternal memory formation, and the maintenance of maternal care [25], [27]. Clinically, this parallels findings in women with correlates with impaired caregiving [28], [27], [29]. The reduction in GABAARα1 mRNA expression observed here is consistent with the work of Gonzalez et al. [30], who reported diazepam-induced downregulation of the GABAA receptor α1 subunit in rat cerebrocortical neuronal cultures [30]. While their findings were derived from in vitro models, our study extends these observations to an in vivo context, demonstrating that prenatal diazepam exposure similarly suppresses GABAARα1 expression in maternally relevant brain regions. This convergence of evidence underscores the broader impact of benzodiazepines on GABAergic signaling across experimental paradigms. Importantly, the concomitant decrease in serotonin levels within the PFC suggests a potential interplay between GABAergic and serotonergic systems in mediating maternal behavior, as both neurotransmitters are known to modulate mood, anxiety, and social interactions.

The impaired maternal behavior observed in this study may reflect a disruption in the balance of inhibitory and excitatory neurotransmission. GABAARα1-containing receptors are pivotal for maintaining tonic inhibition [30], and their downregulation could lead to hyperexcitability in neural circuits governing stress responses, thereby diminishing the dam’s ability to engage in nurturing behaviors. Furthermore, reduced serotonin levels in the PFC—a region implicated in decision-making and emotional regulation—may exacerbate these deficits, as serotonin is critical for affiliative behaviors and stress resilience.

It is important to note that the link between GABAARα1 downregulation and the observed maternal deficits, while supported by our correlative data, remains associative. Future studies are required to establish direct causality. Based on our findings, we hypothesize that the downregulation of GABAARα1 is a key mechanistic step in diazepam-induced impairment of maternal care. A critical test of this hypothesis would be to determine whether targeted restoration of GABAARα1 function for instance, through local microinfusion of a selective agonist or viral vector-mediated overexpression in the mPFC or hippocampus of dams exposed to prenatal diazepam could rescue the deficits in pup retrieval, nursing, and nest-building behaviors. Such experiments would unequivocally define the causal role of this receptor subunit and are a primary objective of our future research.

While our data demonstrate a significant downregulation of GABAARα1 mRNA, a key limitation is the absence of protein-level data. Future studies will utilize western blot analysis to confirm the reduction in GABAARα1 protein and employ immunohistochemistry to determine if prenatal diazepam exposure alters the density or morphology of specific inhibitory neuron subtypes, such as parvalbumin-positive interneurons, in the PFC and hippocampal circuits critical for maternal behavior.

Furthermore, future studies should investigate the neuroanatomical underpinnings of these functional deficits. As demonstrated by da Silva Junior et al. [13] in offspring, the same prenatal diazepam exposure paradigm causes a significant loss of brainstem catecholaminergic and serotonergic neurons [13]. It is therefore a critical next step to determine if similar neuronal loss occurs in dams and contributes to the serotonergic and GABAergic dysregulation we observed. Immunohistochemical analysis of tyrosine hydroxylase (TH) and serotonin (5-HT) positive neurons in key regions like the dorsal raphe nucleus, ventral tegmental area, and within the PFC itself will be essential to establish this link.

Our findings indicate diminished prefrontal serotonin levels in the diazepam-exposed group. This depletion may underlie attenuated maternal responsiveness at a neurochemical level, and inhibition of prefrontal cortex activity markedly compromises both pup retrieval and maternal nesting behavior in lactating rats [31], [32]. Serotonin signaling in the prefrontal cortex plays a critical role in maternal caregiving and neuroplasticity within the maternal brain [32]. Specifically, serotonin regulates maternal aggression and pup-directed licking behaviors through its projections to the medial preoptic area [32], [33]. In addition, da Silva Junior et al. [34] demonstrated that prenatal diazepam exposure alters monoamine concentrations in the brainstem of offspring, which may lead to impaired respiratory control [34]**.**

Diazepam’s disruption of serotonergic transmission—likely mediated by GABAergic inhibition of raphe nuclei—could impair these maternal circuits. In addition to these significant neurochemical alterations, a non-significant trend toward reduced BDNF levels was observed in the hippocampus and prefrontal cortex of diazepam-exposed dams. While this did not reach statistical significance, likely due to the high variability inherent in BDNF measurement, the reduction hints at impaired synaptic plasticity which could compromise long-term maternal behavior. BDNF is regulated by both GABAergic and serotonergic signaling and is critical for maternal behavior [5], [9], [35]. The lack of a significant change may suggest the involvement of compensatory mechanisms or indicate that the primary drivers of the behavioral deficits are the direct disruptions to GABAAR, serotonin, and the HPA axis, with BDNF playing a more modulatory role. Future studies with larger sample sizes and targeted time-course analyses are warranted to fully elucidate the role of BDNF in this model.

## 5 Limitations and future directions

While this study provides valuable insights into postpartum maternal behavior, its narrow focus on immediate postnatal outcomes constrains our understanding of enduring intergenerational consequences. To address this gap, future longitudinal research should track offspring development into adulthood, evaluating potential long-term behavioral, cognitive, or neuroendocrine abnormalities that may arise from perinatal exposures. Expanding this work to include cross-species models (e.g., rodent and human cohorts) could strengthen translational relevance, while integrating multi-omics approaches (e.g., epigenomic, transcriptomic, and proteomic analyses) may uncover biomarkers of risk or resilience. Finally, examining how environmental factors, such as social support or stress, interact with pharmacological exposures could inform personalized interventions to mitigate adverse outcomes across generations. Such interdisciplinary efforts would deepen our understanding of developmental origins of health and disease while guiding clinical strategies for vulnerable populations.

While our findings strongly suggest that the impaired maternal behaviors are a direct result of diazepam's action on the GABAergic system, a key limitation of this study is the lack of pharmacological antagonism to confirm site-specific causality. The gold-standard proof would involve the co-administration of a competitive benzodiazepine antagonist, such as flumazenil, which would be predicted to block diazepam's access to the GABA-A receptor and prevent the observed molecular and behavioral deficits. Future studies designed to include this experimental group are essential to unequivocally confirm that the effects reported here are mediated specifically through the central benzodiazepine binding site.

## 6 Conclusion

In summary, prenatal diazepam administration disrupts maternal behavior and induces region-specific reductions in GABAARα1 mRNA and serotonin within brain circuits critical for caregiving, including the prefrontal cortex and hippocampus. These findings highlight the vulnerability of GABAergic and serotonergic systems to benzodiazepine exposure during pregnancy, mediated by dysregulation of stress-related neurocircuitry—such as hypoactivation of the hypothalamic-pituitary-adrenal (HPA) axis and serotonergic depletion in the prefrontal cortex. Such neurobiological alterations impair stress buffering, emotional regulation, and affiliative behaviors essential for early mother-offspring bonding, raising concerns about the intergenerational risks of benzodiazepine use during gestation. Notably, these preclinical outcomes mirror clinical reports linking perinatal anxiolytic exposure to diminished maternal sensitivity and postpartum bonding difficulties, underscoring the translational relevance of our model. Future studies should investigate whether these behavioral deficits propagate transgenerational effects via altered maternal programming of offspring stress or social reward systems. Additionally, integrating neuroimaging in human cohorts to assess prefrontal cortex-amygdala connectivity and oxytocinergic tone could bridge preclinical mechanisms to clinical phenotypes. Public health initiatives must also address systemic gaps, including improved clinician education on neuroactive medication risks and equitable access to psychosocial support systems for at-risk mothers. By harmonizing mechanistic neuroscience, clinical psychiatry, and policy reform, we can advance perinatal care strategies that safeguard both maternal mental health and neurodevelopmental trajectories across generations.

## Author statement

Hamed Fanaei and Samira Khayat designed the study. Yasaman Moin and Hamed Fanaei carried out the experiments. Hamed Fanaei and Samira Khayat analyzed the data. Hamed Fanaei and Samira Khayat wrote the manuscript.

## CRediT authorship contribution statement

**Yasaman Moin:** Visualization, Software, Project administration, Methodology, Investigation, Data curation. **Hamed Fanaei:** Writing – review & editing, Writing – original draft, Visualization, Software, Project administration, Methodology, Investigation, Funding acquisition, Data curation, Conceptualization. **Samira Khayat:** Writing – review & editing, Writing – original draft, Formal analysis, Conceptualization.

## Ethics approval statement

The study was approved by Ethics Committee of Zahedan University of Medical Sciences (ethical code: IR.ZAUMS.AEC. 1402.001).

## Funding

Financial support for the study was conducted by the Office of Vice-President for Research and Information Technology of Zahedan University of Medical Sciences (code number: 3634).

## Declaration of Competing Interest

The authors declare that they have no known competing financial interests or personal relationships that could have appeared to influence the work reported in this paper.

## Data Availability

Data will be made available on request.
